# Trends in the incidence of peripheral vestibular disorders: a Nationwide population-based study

**DOI:** 10.3389/fneur.2023.1322199

**Published:** 2023-12-12

**Authors:** Shih-Han Hung, Sudha Xirasagar, Luong Huu Dang, Yen-Chun Chen, Yen-Fu Cheng, Herng-Ching Lin, Chin-Shyan Chen

**Affiliations:** ^1^Department of Otolaryngology, School of Medicine, Taipei Medical University, Taipei, Taiwan; ^2^Department of Otolaryngology, Wan Fang Hospital, Taipei, Taiwan; ^3^International Ph.D. Program in Medicine, College of Medicine, Taipei Medical University, Taipei, Taiwan; ^4^Department of Health Services Policy and Management, University of South Carolina, Arnold School of Public Health, Columbia, SC, United States; ^5^Department of Otolaryngology, Faculty of Medicine, University of Medicine and Pharmacy at Ho Chi Minh City, Ho Chi Minh City, Vietnam; ^6^Department of Medical Research, Taipei Veterans General Hospital, Taipei, Taiwan; ^7^Department of Otolaryngology-Head and Neck Surgery, Taipei Veterans General Hospital, Taipei, Taiwan; ^8^Department of Otolaryngology-Head and Neck Surgery, School of Medicine, National Yang Ming Chiao Tung University, Taipei, Taiwan; ^9^Research Center of Data Science on Healthcare Industry, College of Management, Taipei Medical University, Taipei, Taiwan; ^10^School of Health Care Administration, College of Management, Taipei Medical University, Taipei, Taiwan; ^11^Research Center of Sleep Medicine, Taipei Medical University Hospital, Taipei, Taiwan; ^12^Department of Economics, National Taipei University, New Taipei City, Taiwan

**Keywords:** vestibular disorders, peripheral vestibular disorders, epidemiology, incidence, Meniere’s disease

## Abstract

**Purpose:**

This study aimed to examines the long-term trend of incidence of peripheral vestibular disorders between 2010 and 2018 in Taiwan.

**Methods:**

Study-eligible patients were identified from Taiwan’s Longitudinal Health Insurance Database 2005 maintained by the Ministry of Health and Welfare in Taiwan. We retrieved 230,566 patients with a first-time diagnosis of peripheral vestibular disorders between 2010 and 2018. We calculated annual incidence rates of peripheral vestibular disorders per 100,000 population. We used the annual percent change (APC) to test the trend of peripheral vestibular disorders over time.

**Results:**

The mean annual incidence rate of peripheral vestibular disorders during the study period was 1489.6 per 100,000 population. Incidence showed a statistically significant steady decrease from 2010 to 2018 with a mean APC of −6.15% (95% CI = −6.97% ~ −5.32%). The decline was led by Meniere’s disease (APC = −9.83, 95% CI = −10.66% ~ −8.99%), followed by benign paroxysmal positional vertigo (APC = −3.69, 95% CI = −4.53% ~ −3.03%), vestibular neuritis (APC = −7.85, 95% CI = -8.96 ~ −6.73), and other peripheral vestibular dizziness (APC = −5.56, 95% CI = −6.69% ~ −4.43%).

**Conclusion:**

The incidence of peripheral vestibular disorders, overall, and the four major subgroups, benign paroxysmal positional vertigo, Meniere’s disease, vestibular neuritis, and other peripheral vestibular dizziness, all decreased substantially, year by year within the 2010–2018 period.

## 1 Introduction

Vertigo, defined as a sensation of whirling and/or loss of balance, particularly associated with looking down from a great height, is reported to cause a huge burden to the healthcare system, making a significant negative impact on the economy and the community (1, 2). Vestibular disorders are commonly classified as central and peripheral in origin. Approximately 80 percent of vertigo is peripheral, and about 20 percent is due to central nervous system causes. Peripheral vestibular disorders (PVD) are most commonly due to benign processes such as benign paroxysmal positional vertigo (BPPV), while central vertigo is often indicative of more serious pathology (3).

The differences in the underlying pathogenesis often create anxiety and challenges for making the correct diagnosis during first presentation and appropriate management in the early phase. Especially for patients presenting to the emergency department, various protocols are in place to distinguish PVD from central vertigo (4, 5). However, it has been shown that there remains room for improvement in the protocols that do not involve neuroimaging. Reports show that while the absolute frequency of post-discharge stroke following an emergency department visit diagnosed as PVD is extremely low, the relative risk may be markedly higher in certain patient groups, suggesting that some strokes are being misdiagnosed as peripheral vestibular disorders (6, 7). Therefore, understanding the incidence trends of confirmed PVD may be of significant importance for clinicians as it may help physicians to make a more informed clinical judgment in suspecting the most probable type of vertigo and managing patients accordingly.

While several studies are available of the incidence of vertigo, studies examining potential changes in the trends are uncommon (8–10). Under a nationwide population-based study, this study aims to describe the incidence of PVD from 2010 to 2018 in a nationally representative Taiwanese claims database.

## 2 Methods

### 2.1 Database

Data on study-eligible patients were retrieved from Taiwan’s Longitudinal Health Insurance Database 2005 (LHID2005) maintained by the Taiwan Ministry of Health and Welfare. The LHID2005 includes patient data for a period of ten years, from January 2010 to December 2018, including all medical claims data for 2,000,000 National Health Insurance beneficiaries, randomly sampled from all enrollees (*n* = 25.68 million) who were enrolled with the NHI in the year 2010. The Taiwan National Health Research Institutes (NHRI) indicates that based on sample to population comparisons, there is no statistically significant difference between the LHID2005 sample and the population of enrollees on age, gender, and healthcare cost distributions. Therefore, the LHID2005 presents a unique opportunity for Taiwan researchers to carry out large-scale epidemiological investigations on the incidence of diseases.

The study was approved by the Institutional Review Board of Taipei Medical University (TMU-JIRB N202210017) and is in compliance with the Helsinki Declaration. De-identification procedures are used to preserve patient privacy before providing the LHID2010 data to researchers. Patient consent was deemed not necessary to permit the use of de-identified data for research purposes by the NHRI. The Institutional Review Board of Taipei Medical University waived the need for patient informed consent requirement.

### 2.2 Study sample

We identified all patients aged ≥20 years who received a first-time diagnosis of PVD between January 2010 and December 2018, using ICD-9-CM and ICD-10-CM codes for each disease as follows: Meniere’s disease (MD) (ICD-9-CM code 386.0 or ICD-10-CM code H81.0), benign paroxysmal positional vertigo (BPPV) (386.11 or H81.10), vestibular neuritis (VN) (386.12 or H81.2), or other/unspecified peripheral vestibular dizziness (other PVD) (386.10/386.19/386.9 or H81.31/H81.39/H81.9/H83.9). In total, the study included 230,566 patients who had received a first-time diagnosis of the above disorders between 2010 and 2018.

### 2.3 Population data

We retrieved annual population data for the period 2010–2018 from the Registry of beneficiaries (ID) of the LHID2005 and restricted the age group to ≥20 years. We used this denominator to calculate annual incidence rates.

### 2.4 Statistical analysis

All statistical analyses were performed using the SAS statistical package (SAS System for Windows, Version 9.4, Cary NC, United States). We calculated annual incidence rates of peripheral vestibular disorders per 100,000 population by dividing the total cases of new PVD cases during the year by the corresponding year total population aged ≥20 years. We calculated the overall incidence rate as well as incidence rates by sex and type of disorder. We used the annual percent change (APC) to study the trend, a documented measure to characterize disease trends and mortality rates over a pre-specified fixed interval. Two-sided value of *p* of 0.05 was determined statistically significant.

## 3 Results

Of 230,566 patients with a first-time diagnosis of PVDs between 2010 and 2018, 83,494 (36.2%) and 147,072 (63.8%) were males and females, respectively. The mean annual incidence rate was 1489.6 per 100,000 population (standard deviation (SD) = 284.9) during the study period.

**Figure 1:**
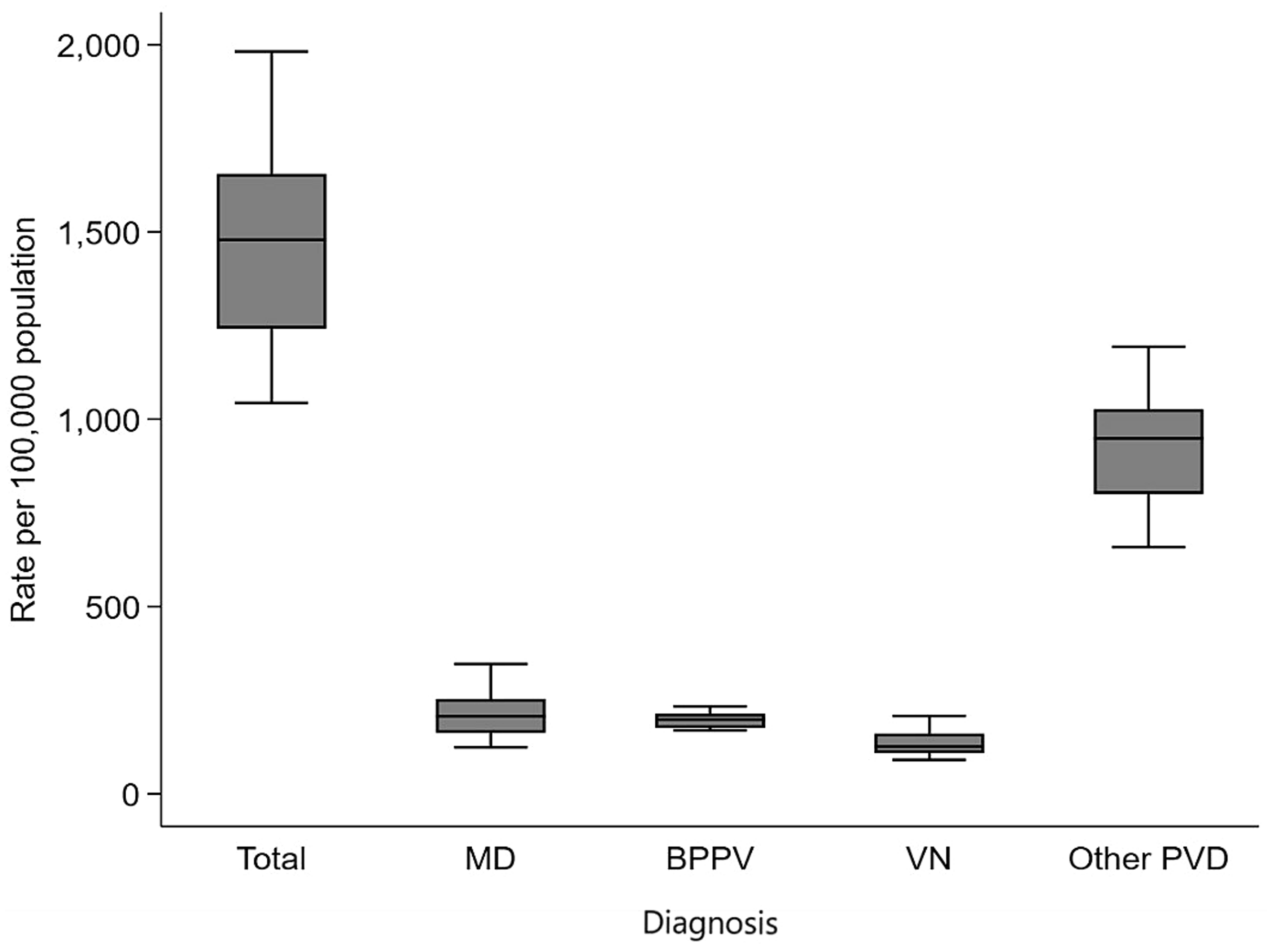
Box plot of data on incidence rates of peripheral vestibular disorders by diagnosis in Taiwan, 2010–2018.

Table 1 presents the mean annual incidence rate of PVDs by type and sex, as well as measures of rate variation over the 9 years period. Over the study years, the mean annual incidence rates per 100,000 population of MD, BPPV, VN, and other PVD were 216.1 (SD = 68.4), 198.5 (SD = 23.1), 136.4 (SD = 35.5), and 938.6 (SD = 161.0) per 100,000 population, respectively (Figure 1). Further, females showed consistently higher mean annual incidence than males for all types of PVDs, MD (270.6 vs. 158.2 per 100,000 population), BPPV (250.1 vs. 143.5), VN (164.6 vs. 106.4), and other PVD (1157.5 vs. 705.4).

**Table 1:** Annual incidence rates of peripheral vestibular disorders per 100,000 population in Taiwan, 2010–2018.

Peripheral vestibular disorders type	Annual Incidence Rate/100,000 populations, 2010–2018			
	Mean annual rate	Standard deviation	Minimum	Maximum
Total	1489.6	284.9	1043.6	1981.7
Males	1113.5	177.3	799.1	1388.8
Females	1842.9	389.6	1277.9	2543.3
Meniere’s disease	216.1	68.4	124.4	346.7
Males	158.2	39.8	95.0	224.4
Females	270.6	96.3	152.5	462.5
Benign paroxysmal positional vertigo	198.5	23.1	169.5	233.5
Males	143.5	12.2	125.0	160.8
Females	250.1	34.0	205.6	302.3
Vestibular neuritis	136.4	35.5	90.7	208.2
Males	106.4	25.8	72.1	157.7
Females	164.6	45.0	108.6	255.9
Other or unspecified peripheral vestibular dizziness	938.6	161.0	658.7	1193.4
Males	705.4	102.3	507.0	845.9
Females	1157.5	218.4	804.0	1522.6

**Figure 2:**
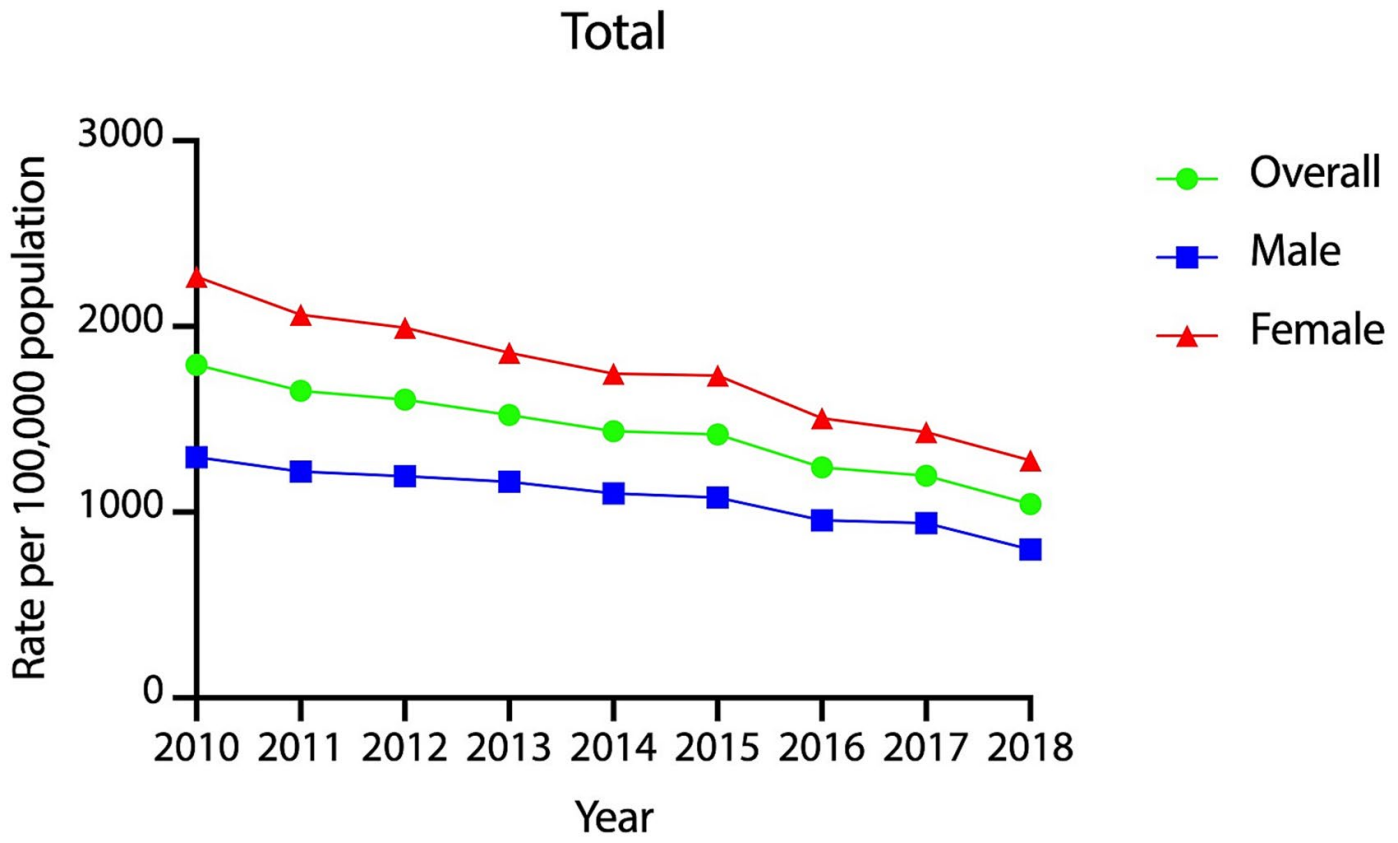
Year-wise incidence rates of peripheral vestibular disorders in Taiwan, 2010–2018.

The year-wise incidence rates of PVDs over the 9 years period are presented graphically in Figure 2. The rates showed a statistically significant, steady decline from 2010 to 2018 with an APC of −6.15% (95% CI = −6.97% ~ −5.32%, *p* < 0.001). Year-wise incidence rates of PVDs were 1793, 1,654, 1,606, 1,522, 1,435, 1,419, 1,242, 1,196, and 1,043 per 100,000 in the years 2010, 2011, 2012, 2013, 2014, 2015, 2016, 2017 and 2018, respectively. Furthermore, the decline was statistically significant and steady among both males (APC = −5.18, 95% CI = −6.18% ~ −4.16%, *p* < 0.001) and females (APC = −6.71, 95% CI = −7.47% ~ −5.95%, *p* < 0.001).

**Figure 3:**
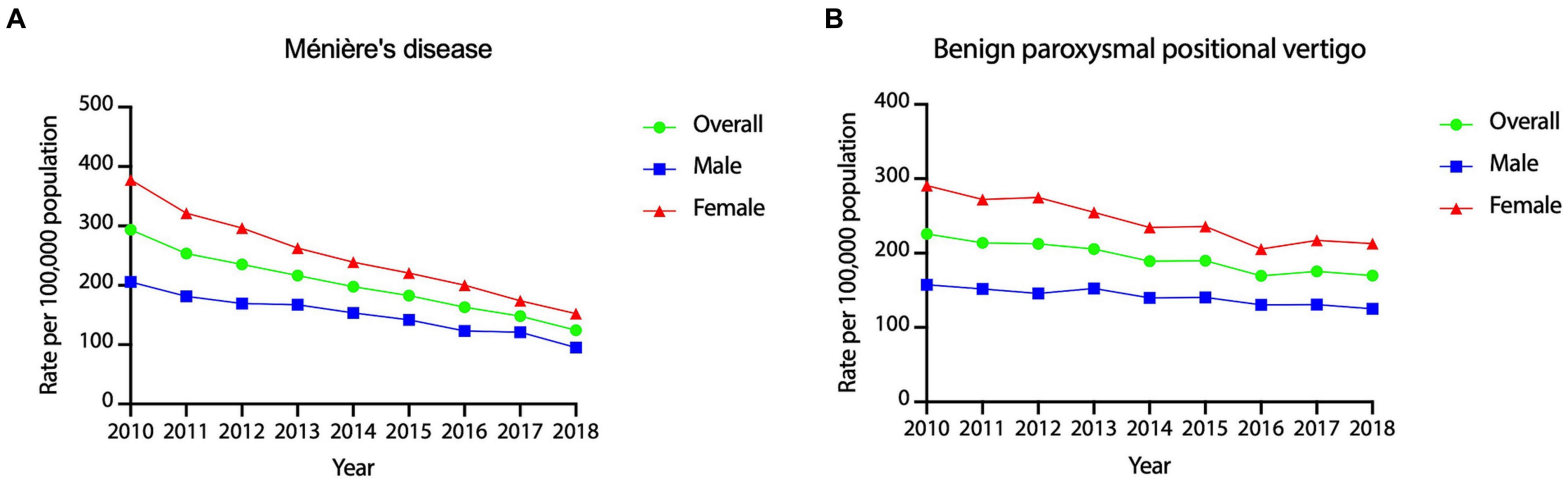
Year-wise incidence rates of Meniere’s disease and benign paroxysmal positional vertigo in Taiwan, 2010–2018.

Figure 3 shows the year-wise incidence rates of Meniere’s disease and benign paroxysmal positional vertigo from 2010 to 2018, showing statistically significant declines, with APC of −9.83% for Meniere’s disease (95% CI = −10.66% ~ −8.99%, *p* < 0.001) and −3.69% for benign paroxysmal positional vertigo (95% CI = −4.53% ~ −3.03%, *p* < 0.001).

**Figure 4:**
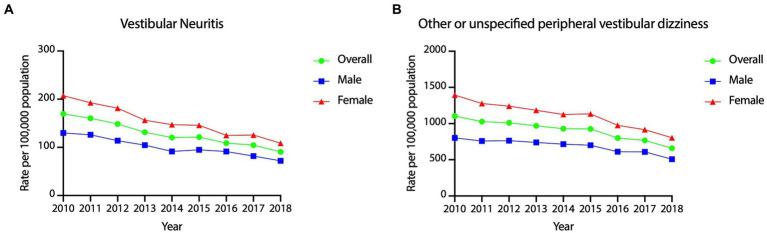
Year-wise incidence rates of vestibular neuritis and other/unspecified peripheral vestibular dizziness in Taiwan, 2010–2018.

The year-wise incidence of vestibular neuritis and other/ unspecified peripheral vestibular dizziness are presented in Figure 4. We found statistically significant declines in vestibular neuritis (APC = −7.85, 95% CI = −8.96 ~ −6.73, *p* < 0.001) and other/unspecified peripheral vestibular dizziness (APC = −5.56, 95% CI = −6.69% ~ −4.43%, *p* < 0.001) over the study period.

## 4 Discussion

This study shows that not only the overall incidence of PVD, but also the four major categories, BPPV, MD, VN, and other PVD, declined year over year from 2010 to 2018 in Taiwan. The findings provide important cues not only for physicians in making their clinical judgments but also for public health policy makers and health services managers.

The average annual incidence of PVDs between 2010–2018 was 1.49% (1489.6/100,000 individuals), which is comparable to a study from Germany reporting their 2015 prevalence rate of peripheral dizziness at 1.6% (1,618/100,000), based on a population-based epidemiological survey (11). Furthermore, our findings showing annual incidence rates of MD and VN at 0.216 and 0.136%, respectively, are similar to the rates found in the same survey (MD prevalence 0.2%; VN 0.16%) among the German population of 70 million,11 by far the largest sample reported in an epidemiological study of vertigo. However, among the four vertigo types, BPPV showed the highest incidence of 0.46% in the German study, compared to 0.198% incidence in our study. BPPV has been generally regarded as the most common peripheral dizziness diagnosis (12, 13). One systemic review of community-based epidemiologic studies of balance disorders showed 1-year incidence estimates of BPPV ranging from 0.06 to 0.6% (14). It appears possible that Taiwan’s rate of diagnosed cases may be lower than actual rates despite Taiwan’s readily accessible and affordable healthcare system under National Health Insurance. The widely used diagnosis method based on the canalith repositioning maneuver (13), and the self-limited nature of BPPV may be causing underestimation of BPPV incidence in the Taiwan population.

Regarding Meniere’s disease, a 30 years epidemiologic and clinical study in Rochester showed that while there was no change in the annual incidence rate from 1951 through 1970, there was a slight decrease from 1971 through 1980 (15). Another smaller-scale study in Taiwan showed a declining incidence of Meniere’s disease (16), consistent with our study. The decline in Meniere’s disease may be due to trends of low-salt diet preference in developed countries where chronic diseases such as diabetes and hypertension are a major concern (17).

Various studies have suggested increased BPPV incidence in women and old adults (18, 19). A negative association between treated osteoporosis and BPPV in women is documented (20). Given that estrogen replacement therapy is shown to reverse BPPV, it is possible that the nationwide popularity of estrogen replacement therapy may be partly causing the declining incidence of BPPV, which however needs to be further investigated (21). There is scant literature on the trend of vestibular neuritis. Given the close relationship of VN with the herpes simplex virus, the declining trend in the seroprevalence of HSV-1 and HSV-2 in the United States might help explain the situation (22). However, there are no long-term data on HSV serology surveys in Taiwan, and further study is needed to identify the causes of decline in VN incidence in Taiwan.

With the observed steady decline in PVDs, there is a need for more investigation of patients with vertigo to seek a central nervous system source as the cause of vertigo, such as atypical strokes. Although the frequency of early stroke after discharge from an emergency department with a diagnosis of a peripheral vestibular disorder is extremely low, Atzema et al. reported that some strokes, or sentinel events for strokes, are being misdiagnosed as PVDs (7, 23). Various protocols have been proposed to improve the differential diagnosis of vertigo in the emergency department. Incorporating computed tomography of the head as a diagnostic aid has provided a low diagnostic yield (2.2, 1.6% for emergency department patients), and addition of MRI changes the diagnosis up to 16% of the time, 8% in acutely ill cases (24). However, it would be impractical and cost-prohibitive for these patients to be evaluated by MRI.

It may be useful to sensitize physicians to the important, often diagnostic value of more detailed physical examinations, including the traditional neurological assessments, and recently developed functional evaluations, such as the head impulse test, before making a diagnosis of peripheral vertigo. If available, MRI may also be considered in higher-risk patients.

To the best of our knowledge, this is the first study to investigate trends in the incidence of dizziness/giddiness with peripheral etiology. However, there are several limitations to this study. First, as the study relied on national health-insurance claims data, vestibular diseases were categorized according to ICD-10-CM. However, due to limitations of the database, it is not possible to accurately retrieve the clinical criteria or guidelines used to determine each participant’s clinical group. Furthermore, as detailed examinations and tests results are unavailable in claims data, diagnoses coded in claims may be provisional or inaccurate which has the potential to introduce bias into the study results. However, if the specific vertigo type was uncertain initially, the patient is most likely to be assigned the diagnosis of “other/ unspecified peripheral vestibular dizziness,” with a shift to a more precise diagnosis at a later point. However, we did not observe an increase in the unspecified category, and the trend of this category followed the trend of every other category, suggesting that provisional diagnosis may have a limited impact on the validity of findings. Second, the declining trend could be attributed to population aging. Since 1993, Taiwan is categorized as an aging society, and aging issues have become a compelling part of the agenda both in policy and medical practice in the country (25). However, changes in aging population would not significantly impact the validity of our findings. The total population aged 65 and older increased about 4–5% from 2010 to 2018, far less than the annual year-on-year decline of peripheral vertigo demonstrated in our study. Finally, it is worth noting that our study did not include vestibular migraine as one of the prevalent peripheral vestibular disorders, given its central involvement. As this condition may be more widespread than both Meniere’s disease and vestibular neuritis, there could potentially be some bias in our research design.

Despite of these limitations, the study findings make a contribution to the literature and have implications for practice. Given that many of the peripheral vestibular disorders are self-limited and can be managed conservatively, physicians may need to prioritize the possibility of central vertigo as the cause in any given patient, given the population-wide, steep decline in the incidence of peripheral vertigo. More aggressive evaluation to rule out central causes and corresponding clinical management may be prioritized if a patient provisionally diagnosed with peripheral vertigo does not respond adequately to the prescribed treatment in a timely manner.

In conclusion, the overall incidence of PVDs, including the four major categories of BPPV, MD, VN, and other PVD, all decreased year on year during the decade of 2010–2018. While the causes of the decline need further investigation, the study findings provide important cues for physicians in making clinical judgments and planning the care of patients with vertigo.

## Data availability statement

The data analyzed in this study is subject to the following licenses/restrictions: Data from the National Health Insurance Research Database, now managed by the Health and Welfare Data Science Center (HWDC), can be obtained by interested researchers through a formal application process addressed to the HWDC, Department of Statistics, Ministry of Health and Welfare, Taiwan (https://dep.mohw.gov.tw/DOS/lp-2506-113.html. 02/01/2022). Requests to access these datasets should be directed to HWDC, Department of Statistics, Ministry of Health and Welfare, Taiwan (https://dep.mohw.gov.tw/DOS/lp-2506-113.html. 02/01/2022).

## Ethics statement

The study obtained approval from the institutional review board of Taipei Medical University (TMU-JIRB N202210017) and is compliant with the Declaration of Helsinki. Patient consent was waived because this study used retrospective data.

## Author contributions

S-HH: Conceptualization, Supervision, Writing – original draft, Writing – review & editing. SX: Writing – original draft, Writing – review & editing. LD: Writing – original draft. YC-C: Writing – original draft. Y-FC: Validation, Writing – original draft. H-CL: Conceptualization, Methodology, Validation, Writing – original draft. C-SC: Data curation, Formal analysis, Methodology, Validation, Writing – original draft.
